# Segmental Liver Transplantation From Living Donors Report of the Technique and Preliminary Results in Dogs

**DOI:** 10.1155/1990/74721

**Published:** 1990

**Authors:** Daniel Cherqui, Jean C. Emond, Andrea Pietrabissa, Mireille Michel, Manuela Roncella, Silas B. Brown, Peter F. Whitington, Christoph E. Broelsch

**Affiliations:** 1 Departments of Surgery and Pediatrics The University of Chicago Pritzker School of Medicine 5841 South Maryland Avenue Chicago Illinois 60637 USA; 2 Service de Chirurgie Hôpital Henri Mondor, 51 Avenue due Maréchal de Lattre-de-Tassigny Créteil 94010 France; 3 The University of Chicago Department of Surgery 5841 S. Maryland Avenue Box #259 Chicago IL 60637 USA

## Abstract

A technique of orthotopic liver transplantation using a segmental graft from living donors was developed in the dog. Male mongrel dogs weighing 25–30 kg were used as donors and 10–15 kg as recipients. The donor operation consists of harvesting the left lobe of the liver (left medial and left lateral segments) with the left branches of the portal vein, hepatic artery and bile duct, and the left hepatic vein. The grafts are perfused in situ through the left portal branch to prevent warm ischemia. The recipient operation consists of two phases: ^1^total hepatectomy with preservation of the inferior vena cava using total vascular exclusion of the liver and veno-venous bypass, ^2^implantation of the graft in the orthotopic position with anastomosis of the left hepatic vein to the inferior vena cava and portal, arterial and biliary reconstruction. Preliminary experiments consisted of four autologous left lobe transplants and nine non survival allogenic left lobe transplants. Ten survival experiments were conducted. There were no intraoperative deaths in the donors and none required transfusions. One donor died of sepsis, but all the other donor dogs survived without complication. Among the 10 grafts harvested, one was not used because of insufficient bile duct and artery. Two recipients died intraoperatively of air embolus and cardiac arrest at the time of reperfusion. Three dogs survived, two for 24 hours and one for 48 hours. They were awake and alert a few hours after surgery, but eventually died of pulmonary edema in 2 cases and of an unknown reason in the other. Four dogs died 2–12 hours postoperatively as a result of hemorrhage for the graft's transected surface. An outflow block after reperfusion was deemed to be the cause of hemorrhage in these cases. On histologic examination of the grafts, there were no signs of ischemic necrosis or preservation damage.

This study demonstrates the technical feasibility of living hepatic allograft donation. It shows that it is possible, in the dog, to safely harvest non ischemic segmental grafts with adequate pedicles without altering the vascularization and the biliary drainage of the remaining liver. We propose that this technique is applicable to human anatomy.


*HPB Surgery*, 1990, Vol. 2, pp. 189–204
Reprints available directly from the publisher
Photocopying permitted by license only
© 1990 Harwood Academic Publishers GmbH
Printed in the United Kingdom

### SEGMENTAL LIVER TRANSPLANTATION FROM LIVING DONORS Report of the Technique and Preliminary Results in Dogs

DANIEL CHERQUI*, JEAN C. EMOND, ANDREA PIETRABISSA, MIREILLE MICHEL, MANUELA RONCELLA, SILAS B. BROWN, PETER F. WHITINGTON and CHRISTOPH E. BROELSCH

*Departments of Surgery and Pediatrics The University of Chicago - Pritzker School of Medicine 5841 South Maryland Avenue Chicago Illinois 60637*

*(Received 16 May 1989)*

A technique of orthotopic liver transplantation using a segmental graft from living donors was developed in the dog. Male mongrel dogs weighing 25–30 kg were used as donors and 10–15 kg as recipients. The donor operation consists of harvesting the left lobe of the liver (left medial and left lateral segments) with the left branches of the portal vein, hepatic artery and bile duct, and the left hepatic vein. The grafts are perfused in situ through the left portal branch to prevent warm ischemia. The recipient operation consists of two phases: [1] total hepatectomy with preservation of the inferior vena cava using total vascular exclusion of the liver and veno-venous bypass, [2] implantation of the graft in the orthotopic position with anastomosis of the left hepatic vein to the inferior vena cava and portal, arterial and biliary reconstruction. Preliminary experiments consisted of four autologous left lobe transplants and nine non survival allogenic left lobe transplants. Ten survival experiments were conducted. There were no intraoperative deaths in the donors and none required transfusions. One donor died of sepsis, but all the other donor dogs survived without complication. Among the 10 grafts harvested, one was not used because of insufficient bile duct and artery. Two recipients died intraoperatively of air embolus and cardiac arrest at the time of reperfusion. Three dogs survived, two for 24 hours and one for 48 hours. They were awake and alert a few hours after surgery, but eventually died of pulmonary edema in 2 cases and of an unknown reason in the other. Four dogs died 2–12 hours postoperatively as a result of hemorrhage for the graft's transected surface. An outflow block after reperfusion was deemed to be the cause of hemorrhage in these cases. On histologic examination of the grafts, there were no signs of ischemic necrosis or preservation damage.

This study demonstrates the technical feasibility of living hepatic allograft donation. It shows that it is possible, in the dog, to safely harvest non ischemic segmental grafts with adequate pedicles without altering the vascularization and the biliary drainage of the remaining liver. We propose that this technique is applicable to human anatomy.

KEY WORDS: Liver transplantation, hepatectomy, live donors

* Present address: Service de Chirurgie, Hôpital Henri Mondor, 51, Avenue due Maréchal de Lattre-de-Tassigny, 94010 Créteil France.

Address correspondence to: Dr Jean C. Emond, The University of Chicago - Department of Surgery 5841 S. Maryland Avenue - Box #259 Chicago Il 60637.

### INTRODUCTION

Orthotopic liver transplantation (OLT) can be life saving for infants and children with severe liver disease. However, many of these patients progress to end stage at a young age when donor organs are relatively unavailable. For example, of 35 children with biliary atresia who died during the process of OLT in one series, 25 died while waiting on the list for lack of a donor [1]. Also, rapidly progressive diseases such as fulminant hepatitis do not allow adequate time to obtain a donor, which increases overall mortality. These considerations have prompted the development of alternative approaches including reduced-size grafts [2–5] and recently dividing one organ to serve two recipients [6]. Although the use of living donors for liver transplantation has been postulated in the literature since the 1960s [7,8] this approach has received little attention in the experimental laboratory and, to our knowledge, has not been attempted clinically. It would represent a further step to reduce the shortage of grafts for young children.

There are, however, several important constraints to using living related liver donors. The donor hepatectomy must be performed with minimal or trivial risk. Adequate vascular pedicles and bile duct must accompany the graft or be fashioned from the recipient's tissues. Finally, the graft must be capable of long term function in the recipient to justify the magnitude of the procedure.

The purpose of this study was to develop, in the dog, a technique of OLT using the left hepatic lobe from a living donor.

### ANIMALS AND METHODS

Male mongrel dogs were used. Donors weighed 25–30 kg and recipients 10–15 kg. All studies were approved by the Animal Experimentation Committee of The University of Chicago Division of the Biological Sciences.

### *Anesthesia*

Anesthesia was induced by intravenous Thiamylal sodium (25mg/kg) and maintained, after endotracheal intubation, with 2:1 nitrous oxide-oxygen mixture with 1% halothane. Pancuronium bromide was administered and animals were artifically ventilated. Electrocardiogram and right carotid blood pressure were continuously monitored. The right external jugular vein was catheterized for infusions and measurements of the central venous pressure. Urine output was monitored by urethral catheterization. Lacated Ringer's solution, dextran 70 in 5% dextrose solution and packed red blood cells obtained from other dogs using standard techniques were used for volume replacement. Blood compatibilities were not assessed. Arterial blood gases were analyzed after induction and at regular intervals and were used to adjust ventilatory support and acid-base status. The hematocrit was checked at hourly intervals and maintained above 30% with transfusions. Temperature was monitored with an intraesophageal thermistor probe and controlled by a heating pad, peritoneal irrigation with warm saline and infusion of warm IV fluids. The same technique of anesthesia was used in the donor and the recipient.

### *Surgical Technique*

### *Donor operation*

The abdomen was entered through a bilateral subcostal incision with mid-line extension to the xiphoid. The graft consists of the left hepatic lobe, comprising the left lateral and left medial segments as shown in Figure 1 [9]. The left hepatic lobe was inspected and the falciform, left triangular and gastrohepatic ligaments were completely divided. The left bile duct usually has a long extra-hepatic segment and joins the common bile duct midway between the liver and the duodenum. The left bile duct was first ligated and divided. Its diameter was usually 1–2 mm. The left branch of the portal vein was then exposed and dissected proximally to the portal bifurcation. Its diameter was usually about 1 cm. Branches to the papillary process of the caudate lobe were ligated and divided. In the dog, the common hepatic artery has a sagittal direction and gives rise, proximal to distal, to the right hepatic and the left hepatic branches and terminates as the gastroduodenal artery (Figure 1). The right gastric artery, which ususally originates from the left hepatic branch, was divided. The common hepatic artery was dissected between the right and left hepatic branches and the gastroduodenal artery was dissected as distal as possible. This dissection was designed to isolate the left hepatic artery in continuity with the gastroduodenal artery without altering the rest of the hepatic arterial supply. As it enters the liver, the left portal pedicle gives rise to a branch destined to the quadrate lobe. This branch was ligated and divided after incision of the Glisson's capsule. The capsule joining the papillary process of the caudate lobe and the left lateral segment was incised and arterial and biliary branches to the caudate were divided. The liver parenchyma was transected along an oblique line running from the fissure between the quadrate lobe and the left medial segment to the left side of the supra-hepatic inferior vena cava (IVC). The left hepatic vein was identified at the upper part of this incision at about 1.5 cm in depth and was dissected free for 1 cm in length. The lower parenchyma was crushed with a Kelly clamp and vessels controlled by electrocautery or ligation with fine silk. This dissection leaves the left lobe attached only by its vascular pedicles (Figure 2). At no moment during the dissection was the graft ischemic.

Figure 3 illustrates the technique used to perfuse the liver *in situ*. The left portal branch was clamped at the level of the portal bifurcation and a 14 French rubber catheter was inserted in it and secured with an umbilical tape and tourniquet. The common hepatic artery was ligated just proximal to the left hepatic artery as was the distal end of the gastroduodenal artery. The left hepatic vein was clamped as distal as possible. Infusion 1 liter of cold Collins solution was then started through the left portal branch by means of the rubber catheter as the left hepatic vein was divided to vent the flush solution. The gastroduodenal artery was divided above the tie to allow back flow of the flush solution through the left hepatic artery. Warm ischemia of the segmental graft was thus prevented. Systemic heparinization was used prior to perfusion because of the risk of bleeding from the cut surface of the remaining liver. The abdomen was closed in 1 layer and the skin sutured with interrupted stitches.

Upon removal the graft was stored in the preservation solution at 0.5°C. A fibrin sealant prepared using cryoprecipitate, thrombin and calcium [10] was spread on the cut surface of the graft.

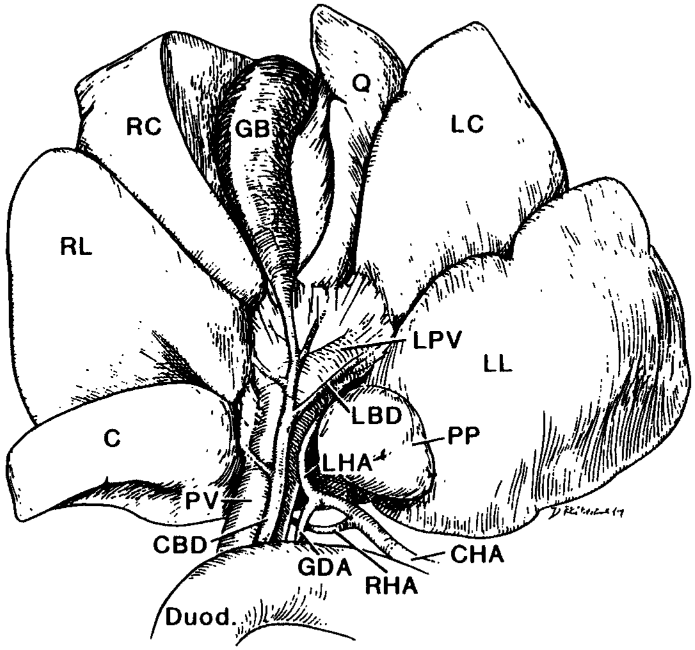

**Figure 1** Liver anatomy in the dog

Duod: duodenum, CBD: common bile duct, PV: portal vein, GDA: gastro-duodenal artery, CHA: common hepatic artery, RHA: right hepatic artery, LHA: left hepatic artery, LBD: left bile duct, LPV: left protal vein.

Liver segments: LL-left lateral, LC-left central, Q-quadrate, RC-right central, RL-right lateral, C-caudate, PP-papillary process of the caudate lobe.

*Recipient Operation*

In addition to the vascular access described above, the left femoral and external jugular veins were isolated for the purpose of veno-venous bypass. The same surgical incision was used. The porta hepatis was dissected as in preparation for routine OLT. The common bile duct and the branches of the hepatic artery were divided as close to the liver as possible. The portal vein was dissected for a distance of 3–4 cm up to the level of its bifurcation and the pancreatico-duodenal vein was ligated and divided. The liver was then completely mobilized by section of its ligaments, and the IVC was dissected free from the retroperitoneum with division of the adrenal vein when necessary.

Spleno-cavo-jugular veno-venous bypass, after splenectomy was used (Figure 4), powered by a centrifugal pump (Biomedicus, Medtronics, Minneapolis, Min) to prevent mesenteric congestion [11]. Total vascular exclusion of the liver was achieved by portal vein occlusion and clamping of the supra-hepatic and infra-hepatic vena cava. Veno-venous by-pass was immediately started and adjusted to achieve proper hemodynamic response and adequate decompression of the portal vein.

Removal of the devascularized liver was begun by ligating and dividing the branches of the portal vein distal to its bifurcation to save as much of its length as

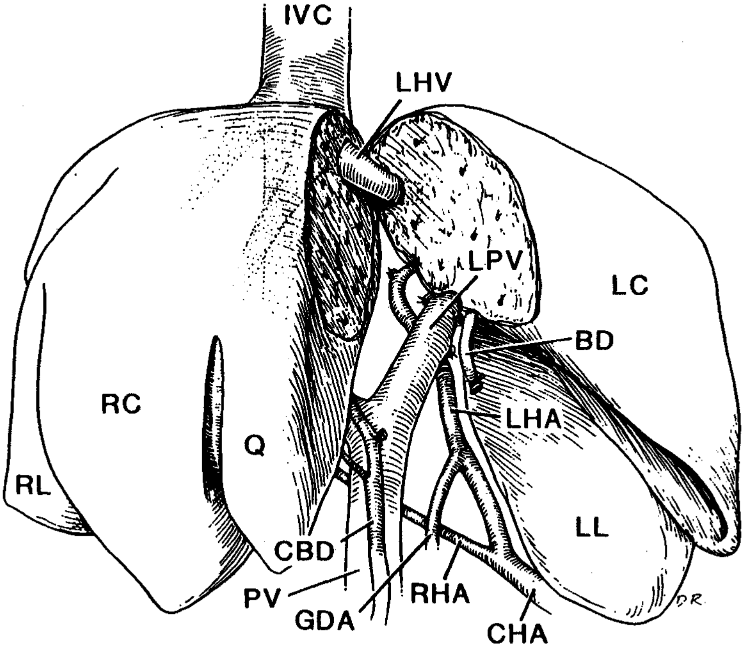

**Figure 2** Dissection of the left lobe graft
IVC: inferior vena cava, LHV: left hepatic vein.

possible. Then the liver was split along the medial fissure dorsally to the level of the IVC. The common trunk of the middle and left hepatic veins was dissected and the bridge between these 2 veins was cut in order to obtain a single larger orifice for hepatic vein anastomosis. On the right side, 3 or 4 large hepatic veins have to be ligated and divided. The bridge of parenchyma between the papillary and caudate processes of the caudate lobe anterior to the IVC was divided. Small hepatic veins draining directly into the IVC from the caudate lobe on both sides were divided. Thus, total hepatectomy was completed while the IVC was preserved (Figure 4). This was achieved without any bleeding due to vascular exclusion. Leaks on the IVC were identified and sutured after releasing the infrahepatic caval clamp while the left hepatic vein was clamped. Fibrin sealant was then spread on the exposed IVC.

Figure 5 shows the implantation of the segmental graft. The hepatic vein of the graft was anastomosed to the common trunk described above using continuous suture of 5/0 polypropylene. A small opening was left on the anterior aspect of the anastomosis to allow flushing of preservation solution from the graft. The portal vein was connected by an end to end anastomosis using 6/0 suture. A 14 French rubber catheter was inserted in the portal vein before the portal anastomosis was completed and the graft was flushed with 500 cc of warm normal saline solution. The portal and hepatic vein anastomoses were then completed. To revascularize the graft, IVC clamps were released, followed by the portal clamp. Arterial

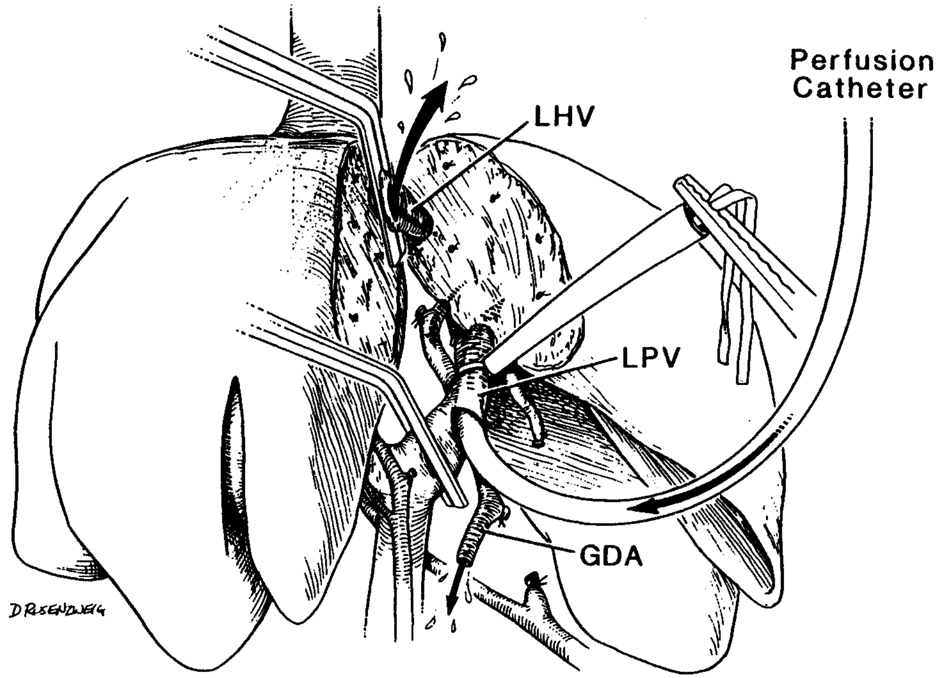

**Figure 3** In situ perfusion of the graft
Note that the catheter was inserted in left portal vein. Outflow of the perfusate without systemic passage was achieved by clamping and opening the left hepatic vein. Ligation of the common hepatic artery prior to its bifurcation and division of the gastroduodenal artery allow backflow of the perfusate in the left hepatic arterial branch.

continuity was restored by anastomosing the distal end of the donor's gastroduodenal artery to the side of the recipient's hepatic artery at the point of bifurcation with the gastroduodenal artery using a running suture of 7/0 polypropylene. Bile drainage was achieved by a duct to duct anastomosis using interrupted stitches of 6/0 polypropylene. The anterior wall of the graft's bile duct was split vertically, when necessary, to increase the diameter of the anastomosis. The abdomen was closed as in the donor.

### *Recovery and Post-Operative Care*

After recovery of spontaneous ventilation, the animals were extubated. The right jugular catheter was tunnelled subcutaneously for purposes of administering drugs and fluids and blood sampling. A solution of 5% dextrose in lactated Ringer's was infused for the first 12 hours and analgesics were injected at regular intervals. Antibiotics (cefazolin 1g/12h) were started at induction and continued postoperatively in donors and recipients. Immunosuppressive therapy consisting of azathioprine (2mg/kg/24h) was started in the recipient after surgery. Normal food was

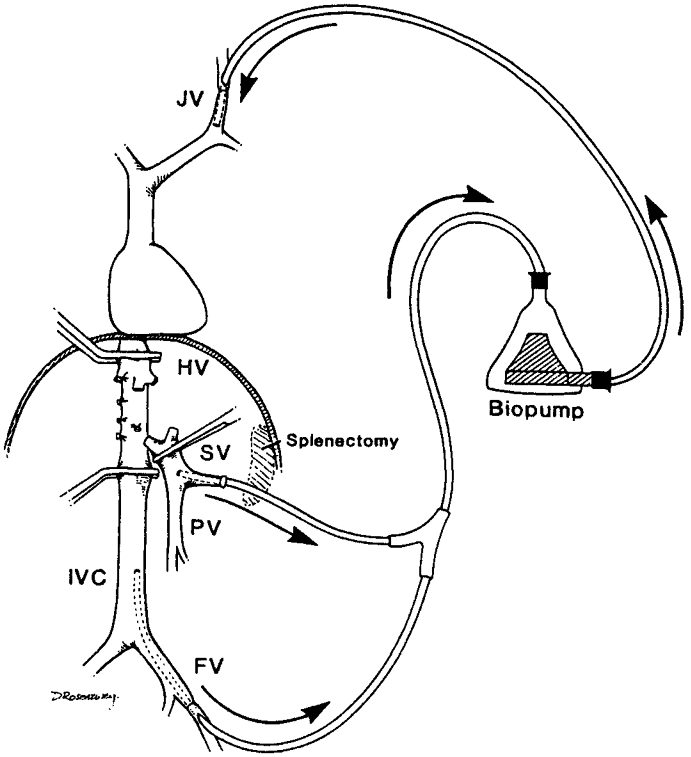

**Figure 4** The recipient after total hepatectomy with preservation of the IVC using veno-venous by pass JV: external jugular vein, SV: splenic vein, FV: femoral vein allowed the day after surgery. Liver function tests (transaminases, bilirubin, alkaline phosphatase and prothrombin time), hematocrit, electrolytes and blood urea nitrogen were measured daily.

### *Experimental Design*

The study was designed in three phases:
Phase 1: To assess the feasibility of the operation, four dogs were autologously transplanted. The left lobectomy was performed as described. Then, the remainder of the liver was resected with preservation of the IVC after a porto-jugular bypass had been instituted. The previously prepared left lobe was autotransplanted in the orthotopic position.
Phase 2: To assess the feasibility of allogenic transplantation by the procedure described, non-survival experiments were undertaken using 9 pairs of dogs. During this phase, the technique was refined to the point that survival experiments could be undertaken.
Phase 3: Survival experiments were performed 10 times as described.

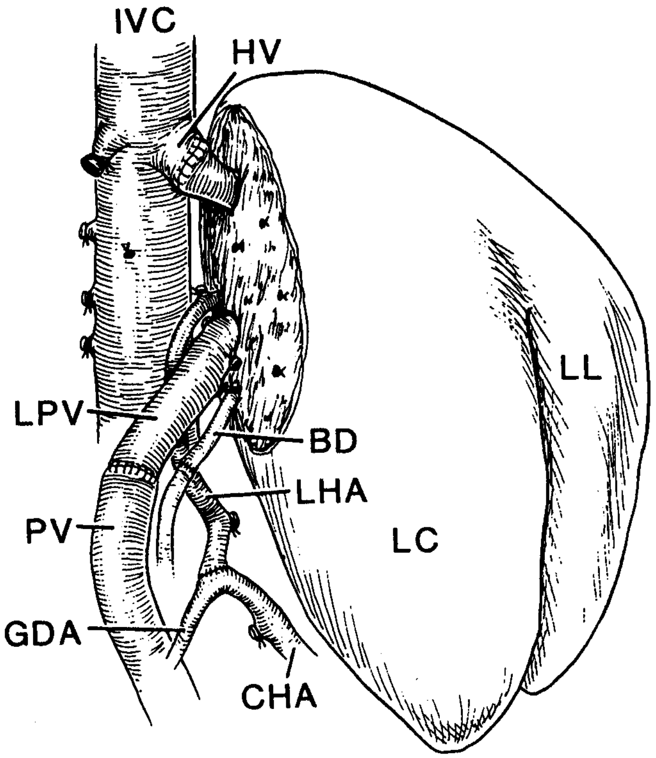

**Figure 5** The graft after implantation in the recipient
The left hapatic vein was directly anastomosed to the inferior vena cava and the lobar vessels and bile duct of the graft are attached to the recipient's main vascular and biliary structures.

### RESULTS

### *Donor Operation*

Mean blood loss in the donors of the phase 3 experiments was 133 ± 98 ml (range 20-300) and no animal required transfusion. The operating time was 3–4 hours. There was no intraoperative death. The first donor died of infected liver necrosis of the quadrate lobe on day 3. After that case, the quadrate lobe and the papillary process of the caudate lobe were always resected. The nine other donor dogs survived without complication. As shown on Figure 6, mild abnormalities of the liver function tests were observed in the postoperative period, but the numbers returned to the normal range within 1 week in all cases. All animals were sacrificed at day 21. Autopsy revealed a subphrenic collection in 1 case and was unremarkable in all other cases except for a hypertrophy of the remaining liver. Liver histology was unremarkable.

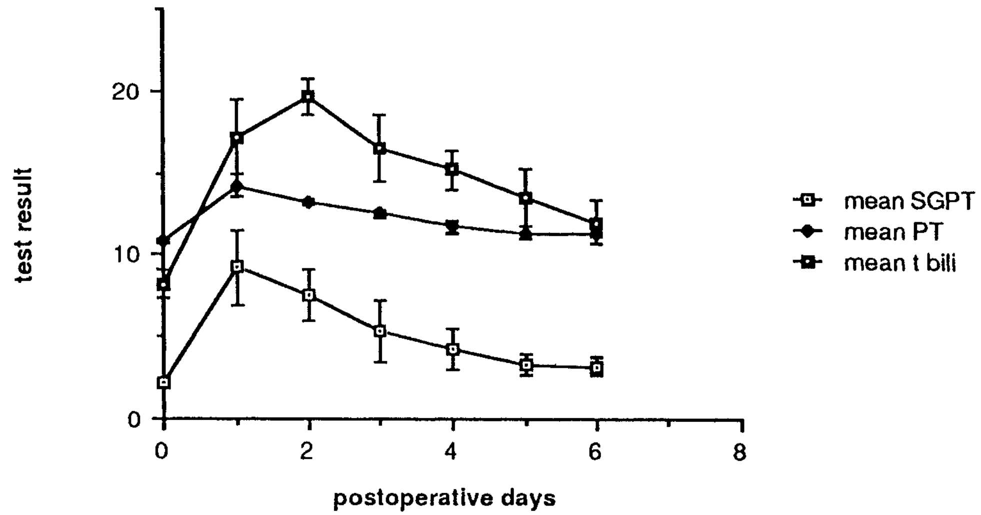

**Figure 6** The changes in SGPT (IU/1/10), prothrombin time (seconds) and total bilirubin (mg/dl x10), as seen in 9 donors after segmental liver resection (mean ± SEM)

In the first 6 cases in phases 1 and 2, the graft was first removed and then flushed *ex vivo,* but this resulted in poor graft appearance. After initiating *in vivo* perfusion as described above, all grafts were completely blanched and soft to touch. *In situ* perfusion did not result in donor reaction that might indicate passage of the potassium rich solution into the systemic circulation. In phase 3 experiments, 9 of 10 grafts were adequate for use in allotransplantation while the other had inadequate vascular pedicles.

*Recipient Operation*

At the beginning of phase 2, a passive portojugular by pass was used with systemic heparinization (100 IU/kg) resulting in uncontrollable hemorrhage after total hepatectomy. The use of the centrifuge pump without heparin and total vascular exclusion of the liver made the total hepatectomy much easier, quicker and bloodless. The hemodynamic tolerance to bypass was excellent. The mean time of venous bypass was 90 ± 16.2 min (range 75-120). No thrombotic complications were encountered.

There were 2 intraoperative deaths in phase 2 due to hemorrhage and 2 in phase 3 resulting from cardiac arrest at reperfusion and air embolus. An outflow block of the graft occurred after reperfusion in 7 instances (3 in phase 2 and 4 in phase 3) causing liver congestion and portal hypertension. this phenomenon has been frequently observed in canine liver transplantation [11,12], but in the setting of segmental liver transplantation it was a particular problem because it led to diffuse bleeding from the graft's transected surface in all cases. The block was reversible in all animals, which indicated the method of hepatic vein anastomosis not to be at fault, but coagulopathy and persistent bleeding were secondarily observed. In the absence of outflow block, revascularization and perfusion of the graft were excellent, and no bleeding from the graft's cut surface was observed.

Mean blood loss and transfusion requirements in phase 3 experiments were 493 ± 432ml (range 150–1300) and 2.14 ± 1.34 units of packed red cells (range 0–4) respectively. The operating time was 3–4.5 hours.

In phase 3 experiments, 2 dogs died intraoperatively. Three dogs survived the immediate postoperative period, two for 24 hours and one for 48 hours. They were awake and alert several hours after surgery, but eventually died of pulmonary edema in 2 cases and of an unknown reason in the other. SGOT were 1500, 1300 and 800 IU/l and prothrombin time was 13, 14.5 and 11.5 seconds respectively one day after transplant. At autopsy there was no hemoperitoneum or ascites, no liver necrosis, bile was present in the bile duct and all anastomoses were patent. Four dogs died 2–12 hours postoperatively as a result of hemorrhage from the graft's transected surface. Persistent outflow block was deemed to be the cause of hemorrhage in these cases, as mentioned above. At autopsy, there was hemoperitoneum without clots, all anastomoses were patent, there was no liver necrosis and there was evidence of bile production in 2. On histologic examination of the grafts, there were no signs of ischemic necrosis or preservation damage (Figure 7).

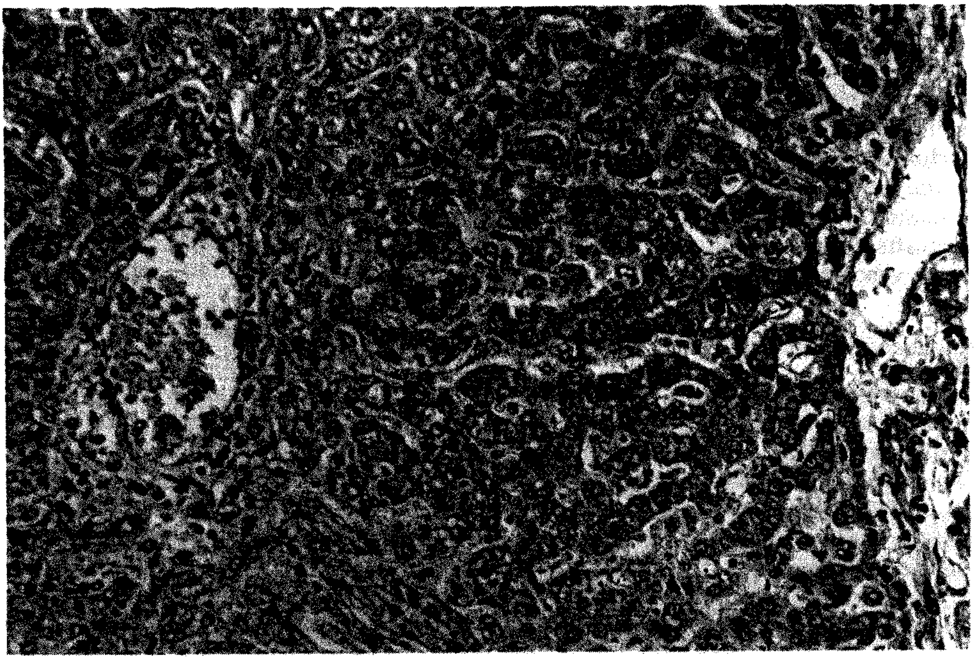

**Figure 7** Histology of post-mortem liver biopsy in a 48-hour recipient survivor
There was no evidence of ischemic necrosis. Central and pericentral regional hepatocytes are well preserved. Portal areas contain a minimal infiltrate consisting primarily of large lymphocytes. There are no vascular lesions. (H & E, × 400)

### DISCUSSION

This study is, to our knowledge, the first report of an attempt at liver transplantation from a living donor. The results show that it was possible, in the dog to: [1] harvest non-ischemic segmental liver grafts with adequate vascular pedicles and bile duct from a living donor without altering the vascularization and the biliary drainage of the remaining liver; [2] perform a total hepatectomy in the recipient with preservation of the IVC; [3] and achieve adequate revascularization of a segmental liver graft using segmental vessels and bile duct and orthotopic hepatic venous anastomosis.

The cornerstones of the technique reported herein are twofold: [1] meticulous dissection of the hilus structures in the donor, identifying all the pedicles and avoiding warm ischemia of the graft, and [2] use of total vascular exclusion of the liver associated with spleno-cavo-jugular venous bypass in the recipient. Although many experimental studies of segmental liver transplantation have used heterotopic and/or auxiliary positioning of the graft [14–19], we chose to use an orthotopic non-auxiliary technique. Because of previous observations that optimal venous drainage of a liver graft was achieved when it was placed as close as possible to the diaphragm [18,20] and that heterotopic grafts can be lost due to inadequate outflow conditions [21], we chose orthotopic positioning. We also avoided auxiliary transplantation by excision of the whole native liver. In other techniques of segmental OLT, the liver parenchyma was reduced in size by an extracorporeal hepatectomy, but the pedicles of the whole liver are used for anastomosis (i.e. IVC, portal vein trunk, celiac trunk and common bile duct) [22,23]. In the present study, segmental portal pedicles and the left hepatic vein were used for reconstruction. The size ratio that we chose between donor and recipient dogs made this possible, as might be the case of a parent providing a hepatic allograft for a child.

The application of this technique to human transplantation depends upon a sufficiently low risk to the donor. In these experiments, donor survival was excellent. The hepatectomy performed for the purpose of supplying a graft is not the same as for the resection of an isolated pathologic process. The vascular pedicles must be retained for both the graft and the remaining liver. It also must include *in situ* perfusion and cooling of the lobe to be resected. This study provides a safe technique to achieve these purposes.

The technique of harvest described herein is applicable to human liver segmental anatomy [24,25] (Figure 8). We have confirmed this possibility by dissections of human cadaver livers. Although left lateral segmentectomy (segments 2 and 3) is certainly the safest formal liver resection, the donor operation should be a left hepatectomy (segments 2, 3 and 4) in order to obtain adequate extrahepatic pedicles and, more importantly, to avoid leaving a compromised quadrate lobe in the donor. Abnormal arterial supply to the liver does not preclude the procedure and can be assessed by a preoperative angiogram. The left hepatic duct has an extra-hepatic segment which can be sewn to a Roux-en-Y intestinal loop. Biliary drainage of part of the right liver in the left hepatic duct exists in 6% of the cases according to Couinaud [24]. Such an anomaly should be sought by a preoperative cholangiogram and considered as a contraindication.

Recent advances in liver surgery have dramatically reduced the operative risk of elective hepatic resections. These advances have permitted hepatic resections with minimal or no blood transfusions. They include mastering of surgical technique,

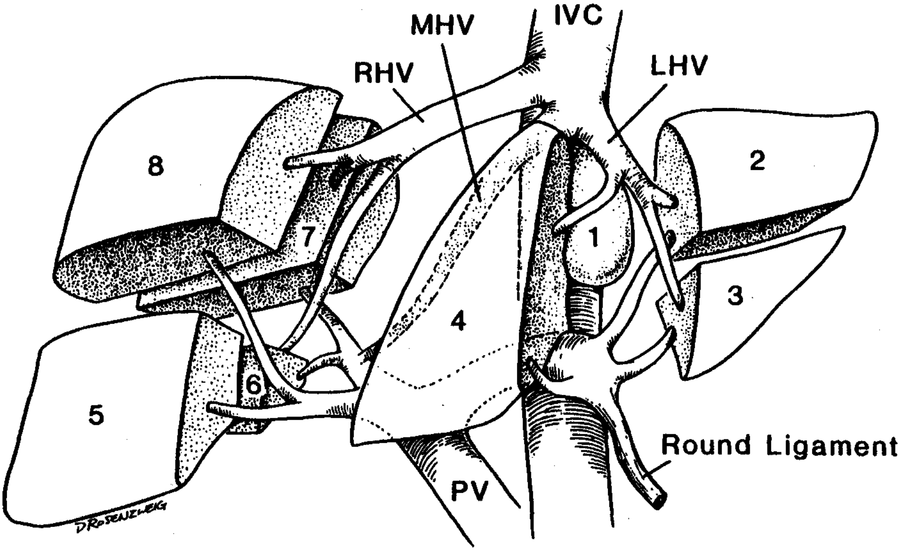

**Figure 8** Segmental liver anatomy in humans according to Couinaud better knowledge of anatomy and new technology such as intraoperative ultrasound and ultrasonic dissectors [26]. Mortality of elective hepatic resections for benign liver lesions, including large hypervascular tumors, has been reported to be as low as 1–2% [26,27]. Elective hepatic resection in a totally healthy patient performed by an expert surgeon can be expected to be performed with an extremely low risk. Morbidity including fluid collections and bile leaks can be avoided by meticulous hemostasis and ligation of small bile ducts. Non specific risks such as thrombo-embolic and septic complications have been accepted in living-related kidney and pancreas transplantation and should not be greater in living liver donors. Liver function has been proven to remain normal after resections of more than 50% of the liver if the parenchyma was normal [28]. The projected resection in living donation does not exceed 35–40% of the liver mass[29]. Moreover, the capacity of the liver for regeneration will permit a full recovery of the hepatic functioning mass [28].

Also required for application to human transplantation is a graft which functions and supports life in the recipient. In these experiments, the recipient survival was poor. This can be ascribed to the extensive recipient operation, which was analogous to human reduced-size liver transplantation and was performed in small dogs. The main cause of death was hemorrhage from the graft's cut surface, which only occurred in those cases where an outflow block of the graft was observed after reperfusion. This problem is specific to the canine model [11,12] and has not been reported in humans or other species. Dogs, which are exquisitely sensitive to portal vein clamping, require complex bypass techniques during the anhepatic phase. In contrast, cirrhotic infants undergoing OLT tolerate prolonged portal and caval clamping without appreciable hemodynamic consequences. Moreover, postoperative care, while intensive by animal experimentation standards, did not approximate that delivered in clinical intensive care units and was insufficient for survival in these animals. However, there was evidence of bile production by the graft in 5 cases, histologic examination of the grafts showed normal parenchyma, and three dogs survived for 24–48 hours and were alert with normal liver function.

Our previous work [3,4], as well as that of others [2,5], have shown that left lobe segmental grafts can result in long term survival with normal liver function in infant hepatic allograft recipients. Bismuth and Houssin have reported a case of auxiliary orthotopic segmental OLT showing that direct anastomosis of the left hepatic vein was possible in humans [30]. Total hepatectomy with preservation of the IVC was perfectly feasible in humans [31,32]. Recently, it has been shown that human cadaver livers can be split into 2 grafts. This has been performed successfully in Germany [6] and in France (H. Bismuth personal communication). We have performed this technique in 7 pairs of patients. Six of 7 infants receiving left lobe grafts without the vena cava are alive with a technique analogous to that of the recipient dogs described in the present study (manuscript in preparation).

There are several potential advantages to the use of living-related hepatic allograft donors in pediatric transplantation. Many children, particularly infants, die without the benefit of transplantation. This mortality can be reduced by using reduced-size allografts, splitting organs for use in 2 recipients, and by living donors. Living related donation may also offer immunologic advantage. Parent to child donation results in at least 1 haplotype HLA identity (except for exceptional translocations). Low stimulation mixed lymphocyte culture (MLC) in nonidentical living related kidney transplantation has been shown to significantly increase the graft survival [33]. Thus, haplotype identity and low stimulation MLC are very likely to increase liver graft survival and should be a prerequisite to consider the procedure. Also, living related donors can undergo extensive preoperative evaluation which would assure normal liver function and avoid graft dysfunction owing to undetected hypotension, drug usage, or other factors often encountered in cadaveric donors. Finally, the procedure could be performed under optimal elective operative conditions.

In conclusion, this study, combined with the clinical experience using reduced liver grafts in children, shows the technical feasibility of living hepatic allograft donation.

### *References*

1. Zitelli BJ, Malatack JJ, Gartner JC, Urbach AH, Williams L, Miller JW, Kirkpatrick B. Evaluation of the pediatric patients for liver transplantation. *Pediatrics* (1986); **78**: 559–565.
2. Bismuth H, Houssin D. Reduced-sized orthotopic liver grafts in hepatic transplantation in children. *Surgery* (1984); **95**: 367–70.
3. Broelsch CE, Emond JC, Thistlethwaite JR, Rouch DA, Whitington PF, Lichtor JL. Liver transplantation with reduced-size donor organs. *Transplantation* (1988); **45**: 519–23.
4. Broelsch CE, Emond JC, Thistlethwaite JR, Whitington PF, Zucker A, Baker AL, Aran PF, Rouch DA, Lichtor JL. Liver transplantation, including the concept of reduced-size liver transplants in children. *Ann Surg* (1988) **208** (4):410–20.
5. De Hemptinne B, de Ville de Goyet J, Kestens PJ, Otte JB. Volume reduction of the liver graft before orthotopic transplantation: report of a clinical experience in 11 cases. *Transplant Proc* (1987); **14**: 3317–22.
6. Pichlmayr R, Ringe B, Gubernatis J, Hauss J, Bunzendahl H. Transplantation einer spender leber auf bei zwei empfanger (splitting transplant). Eine neue methode in der weiterentwicklung der lebersegementtransplantation. *Langenbecks Arch Chir* (1988); **373**: 127–30.
7. Lortat-Jacob JL, Maillard JN, Giuli R, Benhamou JP, Leandri J. Tentatives expérimentales d'autotransplantations de lobes de foie en position heterotopique. *Rev Int Hepat* (1965); **15**: 1491–516.

8. Smith B. Segmental liver transplantation from a living donor. *J Ped Surg* (1969); **4**: 126–32.
9. Sigel B. Partial hepatectomy in the dog. *Arch Surg* (1963); **87**: 788–91.
10. Rousou JA, Engelman RM, Breyer RH.Fibrin glue: An effective hemostatic agent for non suturable intraoperative bleeding. *Ann Thor Surg*(1984); **38**: 409–10.
11. Kam I, Lynch S, Todo S et al. Low flow veno-venous bypasses in small dogs and pediatric patients undergoing replacement of the liver. *Surg Gynecol Obstet* (1986); **163**: 33–6.
12. Starzl TE, Knapp HA, Buck DR, Lazarus RE, Johnson RV. Reconstructive problems with canine liver homotransplantation with special reference to the postoperative role of hepatic venous flow. *Surg Gynecol Obstet* (1960); **111**: 733–43.
13. Eiseman B, Knippe P, Hoh Y, Normell L, Spencer FC. Factors affecting hepatic vascular resistance in the perfused liver. *Ann Surg* (1963); **157**: 532–47.
14. Corry RJ, Chavez Péon F, Miyakimi T, Malt RA. Auxiliary partial liver transplantation in the dog. *Arch Surg* (1969); **98**: 799–802.
15. Reuvers CB, Terpstra OT, Ten Kate FWJ, Koop PPM, JC, Jeekel J. Long term survival of auxiliary partial liver grafts in DLA identical littermate beagles. *Transplantation* (1985); **39**: 113–8.
16. Reuvers CB, Terpstra OT, Boks AL et al. Auxiliary transplantation of part of the liver improves survival and provides metabolic support in pigs with acute liver failure. *Surgery* (1985); **98**: 914–20.
17. Maki T, Slapak M. Can a heterotopically placed segmental liver graft be life supportive? An affirmative finding. *Br J Surg* (1974); **61**: 33–9.
18. Broelsch CE, Then PK, Thistlethwaite JR, Emond JC. Heterotoper oder orthotoper lebersatz durch lebersegmente? *Ergebnisse der Gastroenterologie* (1987); **22**: 66–71.
19. Broelsch CE, Lygidakis N. Experimental segmental auxillary orthotopic liver transplantation. Davis and Geck Film Library 1987.
20. Hess J, Jerusalem C, Van der Heyde MN. Advantages of auxillary liver homotransplantation in rats. *Arch Surg* (1972); **104**: 76–80.
21. Jerusalem C, Van der Heyde MN, Schmidt WJ, Tjebbes FA. Heterotopic liver transplantation: unfavorable outflow conditions as a possible cause for late graft failure. *Eur Surg Res* (1972); **4**: 186–97.
22. Bax NMA, Vermeire BMJ, Dubois N, Madern G, Meradji M, Molenaar JC. Othotopic non auxiliary homotransplantation of part of the liver in dogs. *J Ped Surg* (1982); **17**: 906–13.
23. Rossi G, De Carlis L, Doglia M, Rainer Fassati L, Tarenzi L, Galmarini D. Orthotopic transplantation of partially hepatectomized liver in the pig. *Transplantation* (1987); **43**: 362–65.
24. Couinaud C. le Foie. Etudes anatomiques et chirurgicales. Masson: Paris, 1957.
25. Bismuth H. Surgical anatomy and anatomical surgery of the liver. *W J Surg* (1982); **6**: 3–9.
26. Bismuth H, Castaing D, Garden OJ. Segmental surgery of the liver. In: Nyhus L, ed. Surgery Annual. Norwalk: *Appleton & Lange*, (1988); **20**: 291–310.
27. Iwatsuki S, Shaw BW, Starzl TE. Experience with 150 liver resections. *Ann Surg* (1983); **197**: 247–53.
28. McDermott WV, Ottinger W. Elective hepatic resection. *Am J Surg* (1966); **112**: 376–81.
29. Stone HH, Long WD, Smith RB, Haynes CD. Physiologic considerations in major hepatic resections. *Am J Surg* (1969); **117**: 78–84.
30. Bismuth H. Houssin D. Partial resection of liver grafts for orthotopic or heterotopic liver transplantation. *Transpl Proc* (1985); **17**: 279–83.
31. Calne RY. Surgical aspects of clinical liver transplantation in 14 patients. *Br J Surg* (1969); **56**: 729–36.
32. Ringe B, Pichlmayr R, Lubbe N, Bornscheuer A, Kuse E. Total hepatectomy as a temporary approach to acute hepatic or primary graft failure. *Transpl Proc* (1988); **20**: 552–57.
33. Tiwari JL, Sasaki N. Mixed lymphocyte culture response and graft survival in living-related kidney transplants. In: Terasaki PI, ed. Clinical kidney transplants. Los Angeles UCLA: Tissue Typing Lab, (1985): 227–32

*Accepted by S. Bengmark 15 September 1989*

### INVITED COMMENTARY

The work reported by the liver transplant group at the University of Chicago represents a major conceptual advance in the field of liver transplantation, and is a logical extension of the important work recently reported by this group in providing transplants to two recipients from a single donor. This paper, which addresses the problems of finding livers for paediatric recipients, succeeds in demonstrating the feasibility of living-related organ donation in the area of liver transplantation, a concept which has long been in practice with kidney tranplants, and more recently has been extended to the area of pancreatic grafts.

The authors correctly point out that the limiting factor in the applicability of this technique rests with the ability of the liver team to carry out major hepatic resections with minimal morbidity and virtually no mortality. Improvements in surgical technique have made this eminently possible, and the resection of a left lobe in a healthy adult should carry less hazard than a segmental pancreatic resection which has proven to be acceptable.

A number of questions remain to be answered. The high mortality in the phase 3 recipients is certainly higher than would be expected for dogs receiving whole organ tranplants. In the phase 3 recipients, the mortality rate was 100%. Two dogs died from technical complications of re-perfusion with air emboli, four dogs died in the first 12 hours after transplantation from hemorrhage, and three died between the first and second post-operative days from other causes. While the three late post-operative deaths and the two intra-operative deaths may not be related to problems with the liver itself, the four deaths from hemorrhage are cause for concern. These dogs are described to having had a severe hemorrhagic condition from the cut surface of the liver aggravated by hepatic outflow obstruction. It is possible that the practice of anastomosis of the left hepatic vein to the vena cava may not provide the same free drainage of venous blood as is usually obtained in the whole organ transplant, and that this technique may be prone to the mechanical complications such as kinking of the venous outflow tract. This problem has not been reported in human transplantation using the left hepatic vein as the outflow tract, and may be a problem as the authors state, peculiar to dogs.

The experience in this paper has been limited to donors and recipients whose weight ratios do not exceed 3:1. In the human situation, the parent to child weight ratio may greatly exceed this figure, and make the procedure more difficult because of limitations in available space. The early proponents of the use of reduced-size liver transplants have expressed relectance to exceed weight ratios of greater than 8:1.

The anatomy of the dog liver with its long segment of extra-hepatic left biliary duct and left hepatic artery lends itself more readily to this procedure than the human liver where the technical challenge of anastomosing the small artery and duct in the left lobe may be made more difficult than in the dog because of the short extra-hepatic lengths of these structures.

Before this technique can be safely applied to humans, it may be wise to attempt this procedure in primates or baboons in which the anatomy of the liver and the weight differences between recipients and donors would be more comparable to that likely to be encountered in human transplants.

In addition to solving some of the problems of donor availability for small infants and patients with fulminant hepatic failure, this technique provides for optimal timing in transplantation before recipient deterioration occurs which may jeopardize a successful outcome. The use of living related donor liver transplantation will also allow transplanters to discover if the use of donor antigen pre-treatment by pre-transplant transfusions of donor blood may decrease the severity of rejection and improve long-term results as has occurred in the area of living related renal transplantation and experimental models of liver transplantation in animals.

When this technique is eventually used for human transplantation, the most difficult decision may be the timing of the procedure: when will the risks to the patients of further delays caused by the search for a cadaver donor outweigh the risks to the parent inherent in any major surgical procedure.

Riccardo Superina
University of Toronto
Hospital For Sick Children
Toronto, Canada

